# Treatment of Children's Teeth

**Published:** 1872-05

**Authors:** C. E. Francis

**Affiliations:** New York.


### ARTICLE VII.

### *Treatment of Childrens' Teeth.*

BY C. E. FRANCIS, D. D. S., NEW YORK.

To secure a good healthy set of dentures to the adult, much depends on the care they receive in early life. To be sure, some people who have inherited iron-clad constitutions are fortunate enough to keep their teeth to a good old age; but in these degenerate times such cases are the exception and not the general rule. Comparatively few of the descendants of Adam (particularly in this country) reach their thirtieth year with a full complement of perfect masticators, and many are they who lose half their teeth, or are absolutely toothless before that period. As by each one lost extra labor is imposed upon its fellows, where many are removed those that remain are overtaxed and soon worn out, or from alveolar absorption loosen and fall out. As a result, distorted countenances, constitutions broken down by dyspepsia, and dispositions soured and depressed may be expected ; all of which are dubious blessings to enjoy, and the miserable inheritance to the luckless children born of parents thus afflicted. Admitting, as we must, that a complete set of well behaved dentures is of greater value than a few straggling, rebellious ones, and that for health, comfort, personal appearance, &c., the former condition of these organs should be secured, we as their acknowledged conservators, must bend our energies to accomplish this object.

It would seem to the casual observer that the mouths of young people are beset on every side by their dire enemy known as caries. Each congenital defect or imperfection in structure is a weak point for the intruder to attack. The acidulated secretions lodging in every nook and cranny are steathily searching out all points of easy ingress, and where an advance is actually made the destruction is rapid, unless checkmated by the wisdom and manipulative skill of the dentist; who should be ever on the alert, fully prepared to baffle the encroachments of the mischievous foe.

Much attention should be directed to the preservation of the deciduous teeth. To the frequent inquiry, "Do you approve of filling children's teeth?" I give a most emphatic affirmative reply; and the unanswerable arguments in favor of so doing, when properly stated, cannot fail to convince and convert the most indifferent parent in regard to the benefit gained by this practice. The greatest difficulty I find is to get control of the patient's teeth, or the parent's ear, sufficiently early to insure the best results.

Believing, then, in the importance of retaining the deciduous teeth until their services are no longer needed, and they are forced out of their places by their larger and more substantial successors, our attention should be directed to the latter, with the view of securing to them positions, where, if possible, they may permanently abide, to labor in a good cause, unmolested by torturing forceps, and strangers to the agonizing pangs of merciless odontalgia.

The sixth year molars are usually the first victims to disease, and we are often puzzled to determine just what to do with these teeth, and afford the greatest benefit to our patients. If well calcified and smooth in appearance, the patient blessed with a sound constitution, the dento-maxillary arches well defined, and teeth not crowded, we would at once say, "return them by all means;" but, on the other hand, where they are poorly calcified, the enamel rough and pitted, the teeth badly crowded, with unmistakable evidence of decay all along their approximal faces, the acquired experience and careful observation of years have taught me that they cannot be classed as permanent teeth, and their room is much better than their presence.

Twenty-eight perfect teeth, that can be retained for a life time with comparatively little care, are of greater value than thirty-two badly decayed or even gold-loaded ones, which require constant repairing so long as any vestige of their structure remains. The permanency of entire sets of tolerably good dentures are frequently sacrificed in a fruitless endeavor to retain four sickly, short-lived molars; or, perhaps, rather in adhering too stubbornly to a deep-rooted dogma that it is error to extract a tooth under any circumstances. But in all the varied conditions in which we find these teeth, wisdom and reason should govern us, rather than a fixed rule to retain or extract indiscriminately, to whichever direction our bias may be turned ; and deliberate, careful study of each individual case is often necessary to determine the proper course.

In examining the mouths of young patients, we sometimes find the teeth of a low toned character, showing evidences of some systemic disturbance while in their formative stage. The enamel may be rough and pitted, with deep sulci extending quite through to the dentine, or they may exhibit traces of disintegration on their bucco cervical margins and approximal surfaces. Many of us feel as if this class of teeth were doomed, and we almost despair of their salvation.

The first advisable course of treatment that presents itself to my mind in such cases is, first, to render the rough places as smooth as possible. This may be accomplished by the use of chisels, files, sticks of Superior or Scotch stone, and powdered Arkansas or pumice stone. The burring engine may be made quite useful, both for trimming and polishing the rough surfaces of teeth. Wherever the fissures or pits extend entirely through the enamel, thus exposing the dentine, they should be cut or drilled out and filled. Then the patient needs *thorough* (and I emphasize that word) instruction as to the importance of taking care of these organs, otherwise our efforts to save them may prove futile. They should be taught *how* and where to brush their teeth, so as to be able to remove all extraneous matter from their surfaces; to use soluble dentifrices and alkaline washes; also, to employ phosphates, and to closely observe hygienic laws. If we neglect all this, (as I fear too many do,) we are unfaithful to the trust imposed upon us when such cases are given in our charge.

In filling children's teeth, either deciduous or permanent, much depends on a choice of material used for this purpose. Gold, tin foil, amalgam, oxychloride of zinc, and preparations of gutta percha are called into requisition by different operators. I would rarely recommend gold where the walls of the cavities are soft or deficient in lime salts, but would rather suggest Bevin's or Hill's stopping as temporary fillings; or, perhaps, occasionally tin foil for grinding surfaces. Amalgam and zinc fillings are unreliable in these cases, and I would hardly dare trust them. Tin foil is more readily adapted to this description of cavities than gold, while Bevins' stopping clings to the wall with a determined tenacity, such as no other material possesses. By occasionally watching cavities filled with this substance, and renewing the fillings when much worn away, you may rely upon their safety, which can hardly be said when filled with any other material.

Cases proving this are daily before my eyes, and many of our best operators will testify to the truth of my assertion. The longer I live, and the more closely I observe results, the more thoroughly convinced I am that it is unsafe practice to depend, as many do, wholly upon gold as a filling for young and poorly calcified teeth. Better fill them temporarily with plastic stopping; give them time to become more dense, and at the same time gain the advantage to be derived by operating for patients, who, as the few years are added to their lives, are more easily managed, and for whom our operations may be more satisfactorily performed, with prospects of greater durability, and the better to be appreciated.

Having expressed myself regarding their teeth, permit me a few words about the children who need our care. From what I have stated it may be infered that I believe in the "stitch in time" principle, and I also assent to the old time assertion that "an ounce of prevention is better than sixteen of cure." It is my fortune to have under my charge a great many juvenile patients. I have succeeded in impressing my views concerning their teeth so forcibly upon the minds of parents at such times as they themselves have sought my services, that they send their little ones, often in flocks, for care and advice, and it takes much of my time to attend to them. I generally devote Saturdays and the latter part of nearly every afternoon exclusively to their needs. The most desirable object with me when children make their first visit is to gain their confidence, and this I rarely fail to do, however timid they naturally are. When confidence is once firmly established, it is surprising how easily they can be managed. A smile, a playful remark, a cheerful word, or the exhibition of a toy will have a marvelous effect if carried out in the right spirit. Children study us keenly when first brought into our presence, and it behooves us to create within their minds a favorable impression, for impressions then formed are lasting; indeed, their first idea of the dentist is hardly ever obliterated. If I gain this step, and accomplish nothing more on their first visit, I feel as if I had made a good beginning. To be sure, our patience is at times put to a severe test, but it is bad policy to manifest symptoms of anger or impatience. If tempted to do so, better leave the room for awhile to conquer such feelings, or dismiss the patient at once, with as good grace as possible, offering a word of sympathy. The next time they come they will remember all this, and trusting your friendship will give less trouble. You may say, "Does it pay to be thus bothered with children? Is it not more profitable to whip out their teeth at once, despite all resistance?" I believe it does pay in the "long rnn" to treat them kindly, and even accepting the annoyance, to consider their ultimate good. We must bear in mind the fact that children do not always remain children. As they advance in years their confidence increases, and as they are assured of your faithful endeavors to benefit them, they will ever think of you kindly, and lose no occasion to speak of you to others in words of generous praise.—*Dental Times.*

